# Accuracy of a Standalone Atrial Fibrillation Detection Algorithm Added to a Popular Wristband and Smartwatch: Prospective Diagnostic Accuracy Study

**DOI:** 10.2196/44642

**Published:** 2023-05-26

**Authors:** Jasper L Selder, Henryk Jan Te Kolste, Jos Twisk, Marlies Schijven, Willem Gielen, Cornelis P Allaart

**Affiliations:** 1 Department of Cardiology Amsterdam University Medical Center Amsterdam Netherlands; 2 Department of Epidemiology and Data Science Amsterdam University Medical Center Amsterdam Netherlands; 3 Department of Surgery Amsterdam University Medical Center Amsterdam Netherlands; 4 Regionshospital Nordjylland Hjoerring Denmark

**Keywords:** smartwatch, atrial fibrillation, algorithm, fibrillation detection, wristband, diagnose, heart rhythm, cardioversion, environment, software algorithm, artificial intelligence, AI, electrocardiography, ECG, EKG

## Abstract

**Background:**

Silent paroxysmal atrial fibrillation (AF) may be difficult to diagnose, and AF burden is hard to establish. In contrast to conventional diagnostic devices, photoplethysmography (PPG)–driven smartwatches or wristbands allow for long-term continuous heart rhythm assessment. However, most smartwatches lack an integrated PPG-AF algorithm. Adding a standalone PPG-AF algorithm to these wrist devices might open new possibilities for AF screening and burden assessment.

**Objective:**

The aim of this study was to assess the accuracy of a well-known *standalone* PPG-AF detection algorithm added to a popular wristband and smartwatch, with regard to discriminating AF and sinus rhythm, in a group of patients with AF before and after cardioversion (CV).

**Methods:**

Consecutive consenting patients with AF admitted for CV in a large academic hospital in Amsterdam, the Netherlands, were asked to wear a Biostrap wristband or Fitbit Ionic smartwatch with Fibricheck algorithm add-on surrounding the procedure. A set of 1-min PPG measurements and 12-lead reference electrocardiograms was obtained before and after CV. Rhythm assessment by the PPG device-software combination was compared with the 12-lead electrocardiogram.

**Results:**

A total of 78 patients were included in the Biostrap-Fibricheck cohort (156 measurement sets) and 73 patients in the Fitbit-Fibricheck cohort (143 measurement sets). Of the measurement sets, 19/156 (12%) and 7/143 (5%), respectively, were not classifiable by the PPG algorithm due to bad quality. The diagnostic performance in terms of sensitivity, specificity, positive predictive value, negative predictive value, and accuracy was 98%, 96%, 96%, 99%, 97%, and 97%, 100%, 100%, 97%, and 99%, respectively, at an AF prevalence of ~50%.

**Conclusions:**

This study demonstrates that the addition of a well-known standalone PPG-AF detection algorithm to a popular PPG smartwatch and wristband *without integrated algorithm* yields a high accuracy for the detection of AF, with an acceptable unclassifiable rate, in a semicontrolled environment.

## Introduction

### Screening for Atrial Fibrillation

Atrial fibrillation (AF) is the most common sustained cardiac arrhythmia with a prevalence of approximately 1.5%-2% in the general population. To prevent the most severe AF-related complication, ischemic stroke, AF must be diagnosed first. This can be challenging in individuals with silent paroxysmal AF [1,2]. Many AF-screening studies have been published, and AF yield depends mainly on the duration of screening. The REVEAL AF and CRYSTAL AF trials both showed that prolonged screening resulted in a higher rate of AF detection, the former reporting 6.2% AF detection after 1 month, increasing up to 40% after 30 months of monitoring [3,4].

### Burden Assessment

In addition to diagnosing AF, the assessment of AF burden might be equally important. The LOOP study showed that anticoagulating 70- to 90-year-old patients with one risk factor, who had AF episodes of 6 minutes or more, did not result in fewer strokes or arterial embolisms. In patients of 75-76 years old, the STROKESTOP study found a small net benefit of screening (2 weeks, twice daily, 1-lead electrocardiogram [ECG]) with regard to the primary endpoint, which was a composite of ischemic or hemorrhagic stroke, systemic embolism, bleeding requiring hospitalization, and all-cause death. However, they found no benefit of screening with regard to ischemic stroke alone [5,6]. The NOAH study (non–vitamin K antagonist oral anticoagulant for atrial high-rate episodes of 6 min to 24 h vs standard care) was stopped prematurely due to futility for efficacy [7]. Consequently, a good cutoff for AF burden to initiate anticoagulation to prevent complications such as ischemic stroke still needs to be established.

### Smartwatches or Wristbands

Devices traditionally used for AF diagnosis are not optimal for AF burden assessment: a 12-lead ECG is a point measurement, Holter monitoring can be cumbersome due to wearing discomfort and limited evaluation time, implantable loop recorders are invasive and expensive, and newer techniques such as single-lead ECG devices or photoplethysmography (PPG)–based AF detection apps for smartphones are suboptimal because they are point measurements as well. Commercially available smartwatches and wristbands, however, are often capable of long-term, continuous PPG measurement of the pulsatile blood flow of the wrist. Combining these PPG signals with an AF detection algorithm allows for AF diagnosis as well as AF burden assessment. Several PPG devices using *incorporated* software algorithms have been or are being studied [8-14], but the vast majority of smartwatches or wristbands today do not have an integrated PPG-AF algorithm. Adding a standalone PPG-AF algorithm to these smartwatches and wristbands enables AF detection and burden assessment for these devices without algorithm as well. Furthermore, this creates less dependence on a few high-end and more expensive smartwatch vendors for AF detection. However, accuracy needs to be assessed for all new smartwatch or wristband–standalone PPG-AF algorithm combinations, and this is lacking in literature.

### Objectives

The primary aim of this study was to assess the accuracy of a well-known *standalone* PPG-AF detection algorithm added to a popular wristband and smartwatch, with regard to discriminating AF and sinus rhythm, in a group of patients with AF before and after CV. The secondary objective was to find patient-related predictors of a bad-quality PPG signal.

## Methods

### Study Design, Participants, Data Acquisition, and Reporting

A prospective diagnostic accuracy study was performed. After medical ethics approval, consecutive patients scheduled for electrical CV in the Amsterdam University Medical Center, the Netherlands, were approached for study participation from December 2018 until August 2021. Patients with either atrial flutter or a pacemaker or implantable cardioverter-defibrillator were excluded from the study. During the CV procedure, a first cohort of consenting patients (Biostrap-Fibricheck cohort, December 2018 to February 2020) wore a Biostrap health tracker, and a second cohort (Fitbit-Fibricheck cohort, February 2020 to August 2021) wore a Fitbit Ionic smartwatch. Prior to CV, a 12-lead ECG was obtained, and simultaneously, a measurement set of 5 +/–1 consecutive 1-min PPG measurements was obtained. After CV, a post-CV 12-lead ECG and a similar second PPG measurement set were obtained. The watch or wristband was put on by the nurse without any specific training, and the patient was free to move as normal.

For the Biostrap-Fibricheck cohort, PPG data were sent to the Fibricheck server (Amazon Web Services server in Frankfurt with ISO 27001 certificate) via a Biostrap research application on an Apple iPad. The 1-minute PPG measurements were analyzed in the cloud by the Fibricheck 1.1 algorithm (CE Class IIa PPG-AF detection algorithm, Qompium). In the Fitbit-Fibricheck cohort, the Fibricheck algorithm (version 1.3) was installed on the Fitbit as a clockface, which is a customization of Fitbit smartwatches intended to change the look and feel, and also allowing the use of additional software. The device-software combination was set up to automatically obtain a PPG measurement every other minute, alternating with a minute to transfer the obtained 1-minute measurement to the Fibricheck server (Amazon Web Services server in Frankfurt with ISO 27001 certificate) via the connected smartphone. Subsequently, each 1-minute PPG measurement was automatically analyzed in the cloud by the Fibricheck algorithm.

The Fibricheck algorithm is based on a deep learning model that processes device agnostic PPGs. As a first step, it assesses the 1-minute–PPG waveforms for quality, independent of cause (motion, physiological, or technical induced noise). When quality is deemed insufficient, it reports “insufficiently quality / unclassifiable.” In case of acceptable quality, the next step is rhythm analysis of each 1-minute waveform, which outputs one of the following indicators: (1) *regular sinus rhythm*—regular rhythms with up to 5 isolated ectopic beats per minute; (2) *non-AF arrhythmias*—ectopic beats, tachycardia episodes, and bradycardia episodes; (3) *possible AF*—rhythms with a high likelihood of being atrial fibrillation. Whenever the result was not regular sinus rhythm, the conclusion of the algorithm was reevaluated manually by Fibricheck technicians (blinded for the ECG). For Fibricheck PPG algorithm details, please refer to Selder et al [15], 2020. Measurements in the category “insufficiently quality / unclassifiable” were reported as percentage of the total measurements, but not included in the analysis, comparable to previous analyses [16]. Outcome of the Fibricheck 1-minute PPG analysis was reported on (1) measurement level and (2) measurement set level based on majority vote (MV; the majority of the individual measurement indicators determined the measurement set outcome, and a draw was considered unclassifiable). The outcomes were compared with the (blinded) physician-interpreted 12-lead ECG for both cohorts.

We followed the 2015 standards for reporting diagnostic accuracy (STARD) studies; the list of items is provided in Multimedia Appendix 1.

### Statistical Analysis

Sensitivity (sens), specificity (spec), positive predictive value (PPV), negative predictive value (NPV), accuracy (ACC), and minimal sensitivity (minsens; defined as the sensitivity, with all unclassifiable measurements and AF on the ECG counted as false negative) were assessed for both wearables. Continuous variables are expressed as mean (SD). Categorical data are expressed as counts (percentages). Statistical significance was set at a 2-tailed probability level of <.05. Statistics were performed using IBM SPSS Statistics 27, Python 3.7, and Microsoft Excel 2016.

For the multivariable model, only the contemporary Fitbit-Fibricheck cohort was used. First, univariable logistic generalized estimating equations analyses were performed for all potential predictors. Subsequently, all predictors with a significant univariable *P* value were added to a multivariable model. Starting with this model, a backward selection procedure was used to obtain the final model. The final model consists of only predictors who were significantly related with bad-quality PPG measurements. When collinearity between 2 predictors occurred, the predictor with the lowest *P* value was used in the backward selection procedure.

### Ethics Approval

The Amsterdam University Medical Centre medical ethics board review statement (2018.674) is as follows: “This is a study with a medical device that has already been used in several other studies. The board has no objection to this and issues a non-WMO (Dutch Medical Research with Human Subjects Law, WMO) statement; the reason for this is that the test subjects are not subjected to any action and no behavior is imposed on them, as laid down in the WMO.”

## Results

### Biostrap-Fibricheck Cohort

**Figure 1:**
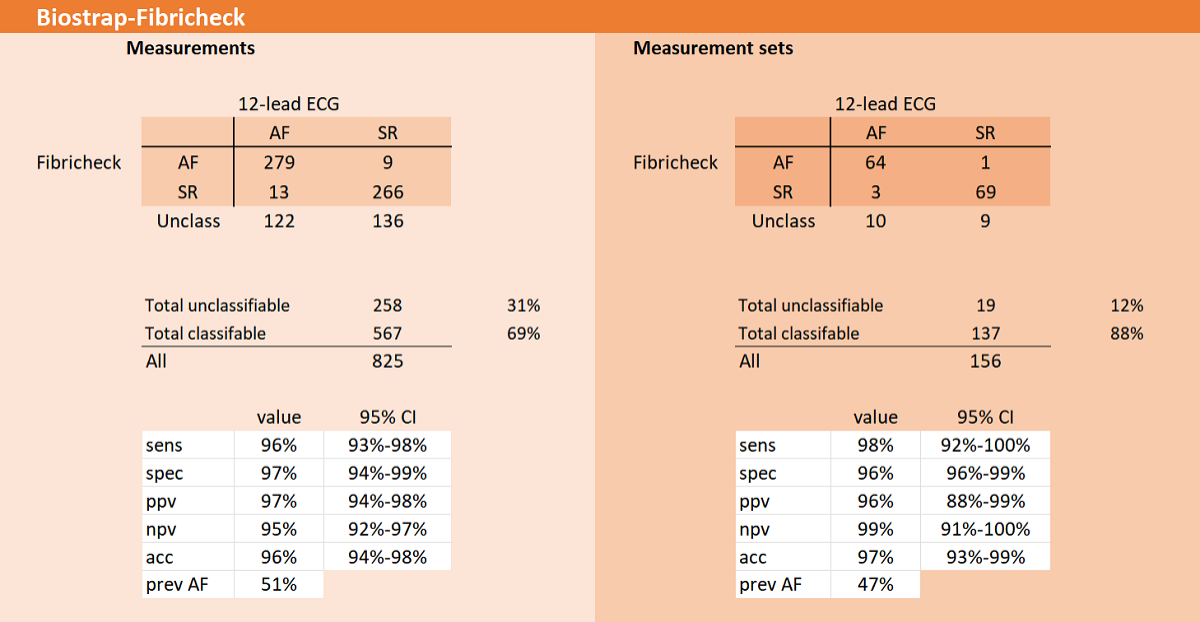
Diagnostic performance for detecting atrial fibrillation in the Biostrap-Fibricheck cohort. acc: accuracy; AF: atrial fibrillation; ECG: electrocardiogram; npv: negative predictive value; ppv: positive predictive value; prev: previous; sens: sensitivity; spec: specificity; SR: sinus rhythm.

In 78 patients with AF, admitted for CV, a total of 825 one-minute PPG measurements (156 measurement sets) were made with the Biostrap wristband and analyzed with the Fibricheck 1.1 algorithm. Baseline characteristics of this cohort are shown in Table 1. Of the 825 PPG measurements, 258 (31%) were not classifiable by the Fibricheck algorithm (bad quality). On measurement set level (MV), this resulted in 19 measurement sets (19/156, 12%) that were not classifiable by the PPG algorithm due to bad quality (Figure 1).

On the measurement level, with a prevalence of AF of 51%, the diagnostic performance of the Biostrap wristband with Fibricheck algorithm for detecting AF after unclassified exclusion was as follows: sens—96 (95% CI 93-98); spec—97 (95% CI 94-99); PPV—97 (95% CI 94-98); NPV—95 (95% CI 92-97); ACC—96 (95% CI 94-98); and minsens—67 (95% CI 63-72). On the measurement set level (MV), with a prevalence of AF of 47%, the diagnostic performance was as follows: sens—98 (95% CI 92-100); spec—96 (95% CI 88-99); PPV—96 (95% CI 88-99); NPV—99 (95% CI 91-100); ACC—97 (95% CI 93-99); and minsens—85 (95% CI 75-92; Figure 1).

**Table 1:** Baseline characteristics of patients in the Biostrap-Fibricheck and Fitbit-Biostrap cohorts.

Variable		Biostrap-Fibricheck (n=78)	Fitbit-Fibricheck (n=73)	*P* value	
Age (years), mean (SD)		65 (9)	67 (10)	.19	
**Sex, n (%)**					
	Male	56 (71.8)	51 (69.9)	.86	
	Female	22 (28.2)	22 (30.1)	.86	
Weight (kg), mean (SD)		86 (17)	88 (16)	.46	
Length (cm), mean (SD)		179 (11)	180 (10)	.56	
**LVEF^a^, n (%)**					
	>52% (normal function)	56 (71.8)	57 (78.1)	.86	
	40%-52% (mild dysfunction)	15 (19.2)	9 (12.3)	.27	
	30%-40% (moderate dysfunction)	4 (5.1)	3 (4.1)	>.99	
	<30% (severe dysfunction)	3 (3.8)	3 (4.1)	>.99	
**Disease or condition, n (%)**					
	Hypertension	39 (50)	34 (46.6)	.74	
	Diabetes mellitus	7 (9)	1 (1.4)	.07	
	Coronary artery disease	7 (9)	5 (6.8)	.76	
	Chronic kidney disease	5 (6)	8 (11)	.39	
	Congestive heart failure	5 (6.4)	6 (8.2)	.76	
	Valvular disease	13 (16.7)	26 (35.6)	.04	
	Stroke	10 (12.8)	6 (8.2)	.43	
	Peripheral vascular disease	2 (2.6)	3 (4.1)	.67	
	Hyperlipidemia	9 (11.5)	19 (26.0)	.03	
	Current smoker	1 (1.3)	5 (6.8)	.11	
**Medication, n (%)**					
	Amiodarone	8 (10.3)	3 (4.1)	.22	
	Flecainide	9 (11.5)	8 (11)	>.99	
	Any beta blocker, including sotalol	54 (69.2)	63 (86.3)	.02	
	Sotalol	19 (24.4)	28 (38.4)	.08	
	Diltiazem	0 (0)	2 (2.7)	.23	
	Verapamil	4 (5.1)	4 (5.5)	>.99	
	Digoxin	3 (3.8)	9 (12.3)	.07	
	ACEI^b^ or AT2^c^ antagonist	27 (34.6)	31 (42.5)	.40	
	Calciumantagonist	13 (16.7)	15 (20.5)	>.99	
	Thiazide diuretics	8 (10.3)	8 (11)	>.99	
	Furosemide	8 (10.3)	9 (12.3)	.80	
**Anticoagulant use, n (%)**					
	OAC^d^	5 (6.4)	5 (6.8)	>.99	
	DOAC^e^	69 (88.5)	68 (93.2)	.40	
	None	4 (5.1)	0 (0)	.12	
CHA_2_DS_2_-VASc-score, mean (SD)		1.9 (1.4)	2.0 (1.5)	.67	
**Number of points with CHA_2_DS_2_-VASc score, n (%)**					
	0	19 (24.4)	10 (13.7)	.10	
	1	14 (17.9)	18 (24.7)	.33	
	≥2	45 (57.7)	45 (61.6)	.74	

^a^LVEF: left ventricular ejection fraction.

^b^ACEI: angiotensin-converting enzyme inhibitor.

^c^AT2: angiotensin II.

^d^OAC: oral anticoagulant.

^e^DOAC: direct oral anticoagulant.

### Fitbit-Fibricheck Cohort

**Figure 2:**
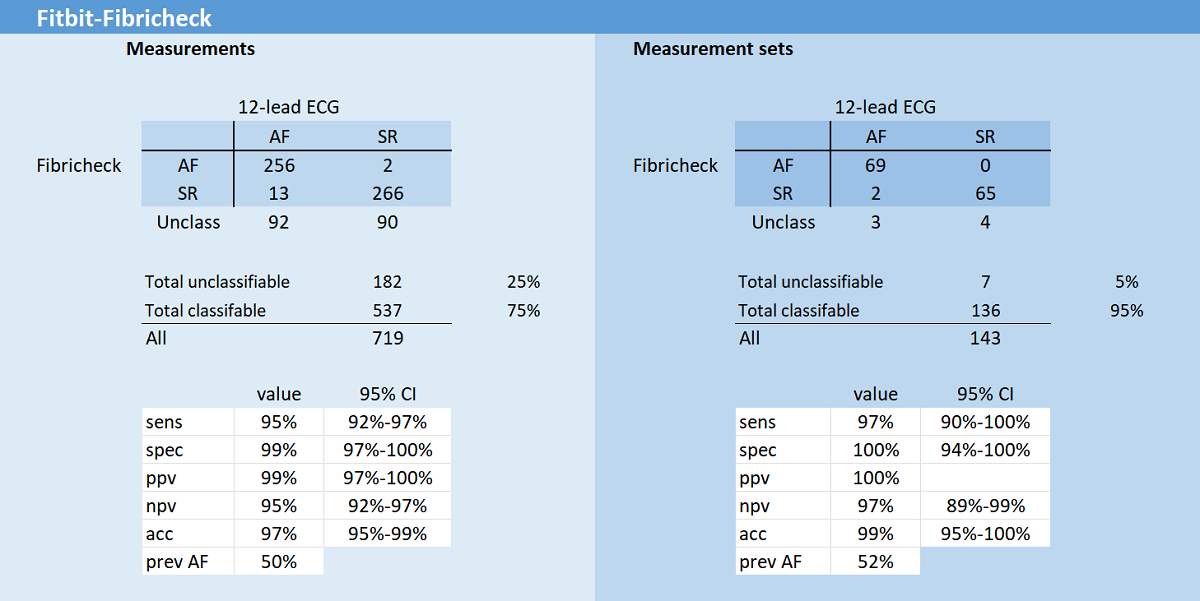
Diagnostic performance for detecting atrial fibrillation in the Fitbit-Fibricheck cohort. acc: accuracy; AF: atrial fibrillation; ECG: electrocardiogram; npv: negative predictive value; ppv: positive predictive value; prev: previous; sens: sensitivity; spec: specificity; SR: sinus rhythm.

In 73 patients with AF admitted for a CV, a total of 719 one-minute PPG measurements (143 measurement sets) were recorded with the Fitbit smartwatch and analyzed with the Fibricheck 1.3 algorithm. Baseline characteristics of this cohort are shown in Table 1. Of these 719 PPG measurements, 182 (25%) were not classifiable by the Fibricheck algorithm because of bad quality. On the measurement set level (MV) this resulted in 7 measurement sets (7/143, 5%) that were not classifiable by the PPG algorithm due to bad quality (Figure 2).

On the measurement level, with a prevalence of AF of 50%, the diagnostic performance of the Fitbit smartwatch with Fibricheck algorithm for detecting AF after unclassified exclusion was as follows: sens—95 (95% CI 92-97); spec—99 (95% CI 97-100); PPV—99 (95% CI 97-100); NPV—95 (95% CI 92-97); ACC—97 (95% CI 95-99); and minsens—71 (95% CI 66-76).

On the measurement set level (MV), with a prevalence of AF of 52%, the diagnostic performance was as follows: sens—97 (95% CI 90-100); spec—100 (95% CI 94-100); PPV—100; NPV—97 (95% CI 89-99); ACC—99 (95% CI 95-100); and minsens—93 (95% CI 85-98; Figure 2).

### Predictors of Bad Quality Measurements

Table 2 shows the results of the univariable logistic generalized estimating equations analyses. After backward exclusion of the nonsignificant predictors, the following patient-related features predicted a Fibricheck bad quality label on the Fitbit-derived PPG measurements: peripheral vascular disease (odds ratio [OR] 10.034, 7.535-13.361; *P*<.001) and direct oral anticoagulant use (OR 2.400, 1.141-5.047; *P*<.02). By contrast, verapamil use predicted significantly less bad quality measurements (OR 0.360, 0.267-0.485; *P*<.001).

**Table 2:** Univariable logistic generalized estimating equations analysis of the Fitbit-Fibricheck cohort (on measurement level).

Feature		Odds ratio^a^	95% CI		*P* value	
			Lower	Upper		
Date of birth		0.995	0.971	1.019	.66	
**Sex**						
	Male	0.739	0.411	1.330	.31	
	Female	1.353	2.433	0.752	.31	
Length		0.997	0.966	1.029	.85	
Weight		0.991	0.974	1.008	.28	
BSA^b^		0.639	0.179	2.278	.49	
**Disease or condition**						
	Hypertension	1.119	0.651	1.922	.68	
	Coronary artery disease	1.481	0.654	3.355	.35	
	Chronic kidney disease	0.771	0.341	1.743	.53	
	Heart failure	1.675	0.693	4.052	.25	
	Valvular heart disease	0.777	0.448	1.348	.37	
	Stroke	2.426	1.000	5.886	.05	
	Peripheral vascular disease	11.035	8.170	14.676	<.001^c^	
	Hyperlipidemia	1.150	0.590	2.423	.68	
	Current smoker	1.003	0.463	2.169	.995	
	Normal ejection fraction	0.776	0.374	1.608	.495	
**Medication**						
	Amiodarone	0.774	0.166	3.609	.74	
	Flecainide	0.992	0.325	3.031	.99	
	Sotalol	1.144	0.655	1.998	.64	
	Diltiazem	1.003	0.715	1.406	.99	
	Verapamil	0.366	0.273	0.490	<.001^c^	
	Betablocker (any)	1.036	0.601	1.786	.90	
	Digoxin	0.876	0.529	1.449	.61	
	OAC^d^	0.419	0.200	0.879	.02	
	DOAC^e^	2.387	1.137	5.009	.02^c^	
	ACE inhibitor	0.971	0.560	1.683	.92	
	Dihydropyridine Ca-antagonist	0.704	0.355	1.397	.32	
	Thiazide diuretics	1.565	0.717	3.417	.26	
	Furosemide	1.859	0.851	4.063	.12	

^a^Odds ratio of >1 predicts a Fibricheck bad quality label on the Fitbit-derived photoplethysmography measurements.

^b^BSA: body surface area.

^c^Significant after backward selection procedure (multivariable analysis). See text for multivariable odds ratios.

^d^OAC: oral anticoagulant.

^e^DOAC: direct oral anticoagulant.

## Discussion

### Principal Findings

This study demonstrates that the Fibricheck standalone algorithm added to 2 popular PPG wrist devices (Biostrap wristband and Fitbit smartwatch) yields a high sensitivity and specificity for detecting AF, with an acceptable unclassifiable or bad quality rate, in a semicontrolled environment before and after a cardioversion procedure. Sensitivity, specificity, and bad quality or unclassifiable rate of this study are comparable to those in previous studies [17]. However, in contrast to most previous studies, mostly evaluating smartwatches and wristbands with *incorporated* PPG-AF algorithms, this study used a standalone PPG-AF algorithm, which can be used in combination with any PPG wristband or smartwatch.

### Comparison of the Two Cohorts

The Biostrap-Fibricheck wristband cohort and the Fitbit-Fibricheck smartwatch cohort show comparable accuracy. However, in the Fitbit-Fibricheck cohort, less measurements and less measurement sets were classified as bad quality (31% vs 25% and 12% vs 5%, respectively). This might be the result of the more extensive training of the algorithm; the Fitbit-Fibricheck cohort was included ~2 years later than the Biostrap-Fibricheck cohort and used a newer version of the Fibricheck algorithm (version 1.3 vs version 1.1). Alternatively, hardware differences between in the 2 wrist devices may have caused the different unclassifiable rate.

### Bad Quality and Its Predictors

The quality check is a necessary first step of the Fibricheck algorithm prior to actual rhythm analysis. Bad quality measurements, typically caused by noise (eg, movement, external light, and electrical interference), need to be filtered out to preserve a high specificity and thus a low number of false positives. Depending on the strictness of the PPG-AF algorithm, around 30% of the single PPG measurements are considered unclassifiable. However, this is likely not a problem as the PPG technique allows for continuous data acquisition, and the unclassifiable rate of multiple measurements combined is much lower. On the other hand, the data in this study were acquired in a semicontrolled setting; using PPG data acquisition in real life might result in a poorer quality and needs to be evaluated. Specific patient characteristics might also evoke unclassifiable PPG measurements. Multivariable analysis of the present data set revealed peripheral vascular disease (OR>10) as the strongest predictor for bad quality, whereas verapamil use (OR 0.366) predicted good-quality PPG measurements. This is likely to be explained by poor peripheral blood flow in peripheral vascular disease, and hence a poor reflected light signal to be analyzed, and the opposite when using the vasodilator verapamil. The third significant parameter contributing to bad-quality PPG measurements, direct oral anticoagulant use (OR 2.4), might be related to increased CHA₂DS₂-VASc, reflecting advanced age and substantial comorbidity.

### Limitations of This Study

There are several limitations to this study. First, in this study, the detection of AF in a single 1-minute measurement was determined by the algorithm, whereas in a measurement set, it was arbitrarily defined by the majority vote (ie, more than half of the measurements in a set should be designated AF, after the exclusion of bad-quality measurements). Although specificity for detection of AF in a single measurement is high, it is presently unknown what cutoff should be used for repeated measurements, especially with high measurement numbers resulting from long-term assessment. Second, this study was performed in a semicontrolled setting, yielding acceptable numbers of bad-quality measurements. These numbers may change in a real-world setting. Third, the prevalence of AF in this study was close to 50%. As PPV and NPV are determined by prevalence, these will be different in different study populations. Finally, we only tested 2 hardware-software combinations; the results might be different for other hardware-software combinations.

### Conclusion and Future Perspective

This study demonstrates that a standalone PPG-AF detection algorithm added to a smartwatch and wristband *without integrated algorithm* yields a high accuracy for the detection of AF in a semicontrolled setting. Combined with the growing smartwatch or wristband market [18] and the fact that a vast majority of these wrist devices do not have an integrated algorithm, this might open new possibilities for AF screening and burden assessment on a global scale.

## Appendix

Multimedia Appendix 1 Standards for reporting diagnostic accuracy (STARD) 2015 checklist.

## Abbreviations

ACC: accuracy
AF: atrial fibrillation
CV: cardioversion
ECG: electrocardiogram
minsens: minimal sensitivity
MV: majority vote
NPV: negative predictive value
OR: odds ratio
PPG: photoplethysmography
PPV: positive predictive value
sens: sensitivity
spec: specificity
STARD: standards for reporting diagnostic accuracy

## Data Availability

The data sets generated or analyzed during this study are available from the corresponding author on reasonable request.
